# Interaction between Single Nucleotide Polymorphism and Urinary Sodium, Potassium, and Sodium-Potassium Ratio on the Risk of Hypertension in Korean Adults

**DOI:** 10.3390/nu9030235

**Published:** 2017-03-05

**Authors:** Yeong Mi Park, Chang Keun Kwock, Kyunga Kim, Jihye Kim, Yoon Jung Yang

**Affiliations:** 1 Department of Foods and Nutrition, College of Natural Sciences, Dongduk Women’s University, Seoul 02748, Republic of Korea; always.blue.spring@gmail.com (Y.M.P.); eluzai81@gmail.com (J.K.); 2 Nutrition and Diet Research Group, Korea Food Research Institute, Seongnam-Si, Kynggi-Do 13539, Republic of Korea; kwock@kfri.re.kr; 3 Statistics and Data Center, Research Institute for Future Medicine, Samsung Medical Center, Seoul 06351, Republic of Korea; kyunga.j.kim@samsung.com; 4 Department of Digital Health, Samsung Advanced Institute of Health Sciences and Technology, Sungkyunkwan University, Seoul 06351, Republic of Korea

**Keywords:** CSK, gene-diet interaction, hypertension, blood pressure, sodium, potassium, sodium-potassium ratio, nutrigenetics

## Abstract

Hypertension is a complex disease explained with diverse factors including environmental factors and genetic factors. The objectives of this study were to determine the interaction effects between gene variants and 24 h estimated urinary sodium and potassium excretion and sodium-potassium excretion ratios on the risk of hypertension. A total of 8839 participants were included in the genome-wide association study (GWAS) to find genetic factors associated with hypertension. Tanaka and Kawasaki formulas were applied to estimate 24 h urinary sodium and potassium excretion. A total of 4414 participants were included in interaction analyses to identify the interaction effects of gene variants according to 24 h estimated urinary factors on the risk of hypertension. CSK rs1378942 and CSK-MIR4513 rs3784789 were significantly modified by urinary sodium-potassium excretion ratio. In addition, MKLN rs1643270 with urinary potassium excretion, LOC101929750 rs7554672 with urinary sodium and potassium excretion, and TENM4 rs10466739 with urinary sodium-potassium excretion ratio showed significant interaction effects. The present study results indicated that the mutant alleles of CSK rs1378942 and CSK-MIR4513 rs3784789 had the strongest protective effects against hypertension in the middle group of 24 h estimated urinary sodium-potassium excretion ratio. Further studies are needed to replicate these analyses in other populations.

## 1. Introduction

Hypertension is widely known to cause cardiovascular diseases including myocardial infarction, congestive heart failure, peripheral vascular disease, stroke, and coronary artery disease [1], which are strongly related to mortality worldwide [2]. Hence, the prevention of hypertension is a vital issue for public health. The prevalence of hypertension is 28.9% (31.8% in men, 26.2% in women) in Korean adults, and is higher in elderly groups over 65 years old (60.5% total, 54.3% in men, 65.0% in women) [3]. The control of blood pressure to prevent hypertension is not a simple undertaking because the mechanism of hypertension is complicated and is related to environmental factors and genetic factors. 

There have been several studies that examined the relationship between single nucleotide polymorphism (SNP) and blood pressure or hypertension via genome-wide association studies (GWAS) among Koreans [4,5,6,7,8,9,10,11,12]. However, the mechanism of blood pressure is not sufficiently explained by the genetic effect. Sodium intake and sodium-potassium ratio are generally known to have positive associations with blood pressure and hypertension [13,14,15,16,17,18,19,20], while potassium intake is known to have a negative association with blood pressure and hypertension [13,21]. The average sodium intake is 3874.1 mg/day in Koreans [3], which is much higher than the 2000 mg/day recommended by the world health organization (WHO) [22]. Urinary sodium is considered to reflect actual ingested sodium as a gold standard [23], but there are difficulties in monitoring it due to extended time and cost in large-scale population studies. The Tanaka equation [24] and the Kawasaki equation [25] are generally used to estimate 24 h urinary sodium or urinary potassium [26,27,28,29] by using spot urinary creatinine, urinary sodium, or urinary potassium.

The protective effect of low sodium diets differs by ethnicity and by individuals according to salt sensitivity [30]. Therefore, research to examine the interaction effects between sodium intake and genetic effects on the risk of hypertension is needed. There was a previous study of this relationship in Koreans, but the study had a small sample size [12]. Therefore, the objectives of this study were to investigate the interaction effects of gene variants and 24 h estimated urinary sodium and potassium excretion and sodium-potassium excretion ratios on the risk of hypertension.

## 2. Methods

### 2.1. Study Population

**Figure 1:**
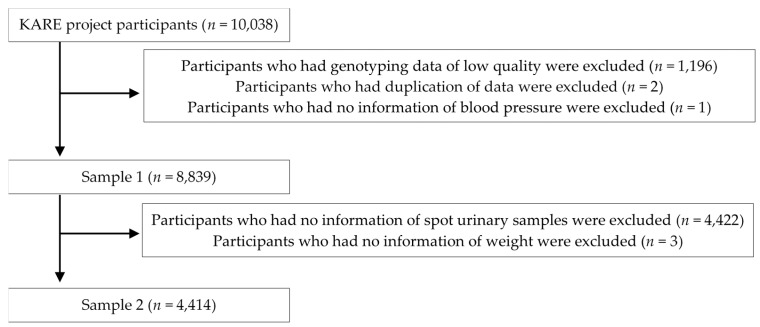
Selection of study participants.

As a part of the Korean genome and epidemiology study (KoGES) cohort, KoGES_Ansan and Ansung study was initially established to investigate the effects of environmental factors on the incidence of chronic diseases among Korean adults in 2001. The KoGES_Ansan and Ansung study primarily included 10,038 participants (5020 in Ansan city and 5018 in Ansung city). Figure 1 shows a flow chart explaining the selection of study participants. Detailed descriptions of the selection process for the study population are below:

**Sample 1**: After excluding participants who had genotyping data of low quality (*n* = 1196) [31], duplicate data (*n* = 2), missing blood pressure data (*n* = 1), sample 1 included 8839 participants. Sample 1 was analyzed to identify SNPs associated with hypertension. 

**Sample 2**: After excluding participants from sample 1 who had no information for spot urinary samples (*n* = 4422) and weight (*n* = 3), sample 2 included 4414 participants. Sample 2 was analyzed to clarify the association between urinary factors and blood pressure or hypertension, and to investigate interaction effects between urinary factors and tagging SNPs on the risk of hypertension.

This study was approved by the electronic institutional review board (e-IRB) of the Korea National Institute for Bioethics Policy (KoNIBP).

### 2.2. Anthropometric Measurement and Collection of Urinary Samples

Information about the participants’ socio-demographic status, medical history, diet, and lifestyles was collected through interviewer-administered questionnaires. Height (cm) was measured by using a stadiometer and weight (kg) was measured by using an audiometer. Waist circumference (cm) was measured three times by using a tapeline and the average values were used. Body mass index (BMI, kg/m^2^) was calculated by using weight and height. 

Measurement of blood pressure (BP, mmHg) was performed three times with a stethoscope using the Korotkoff method in a supine position. We calculated the average value of systolic BP (SBP) and diastolic BP (DBP). If participants had a systolic BP ≥140 mmHg, a diastolic BP ≥90 mmHg, or took medicine for BP, then we defined them as cases with hypertension (1005 cases and 3412 controls).

Urinary specimens were self-collected by the participants in urine cups, then were transferred to conical tubes and sent to a central laboratory. Spot urine tests for sodium and potassium were quantified through biochemical assays in the central laboratory (Seoul Clinical Laboratories, Seoul, Korea). Twenty-four hour estimated urinary excretion of sodium and potassium were calculated using spot urinary sodium and potassium by using the Tanaka formula [24] and the Kawasaki formula [25], which were often used in other studies [26,27,28,29]. We calculated the 24 h estimated sodium-potassium excretion ratio by using 24 h estimated urinary sodium and potassium excretion.

### 2.3. Genotyping and Imputation of SNPs

Comprehensive genotyping, quality controls, and imputation of SNPs have been described in a previous study [31]. Genomic DNA samples were isolated from the blood specimen of participants in the KoGES_Ansan and Ansung study and genotyped using Affymetrix Genome-Wide Human SNP array 5.0 (Affymetrix, Inc., Santa Clara, CA, USA). A total of 500,568 SNPs were genotyped by using Bayesian Robust Linear Modeling Mahalanobis (BRLMM) distance algorithm [32]. SNPs with missing genotype call rates >5%, minor allele frequency (MAF) <0.01, and Hardy-Weinberg equilibrium *p* < 1 × 10^−6^ were eliminated before association analyses. Quality control was conducted by duplicate genotyping for about 1%–2.5% of samples. Imputation of missing genotypes was accomplished with IMPUTE program [33,34] using the Japanese individuals in Tokyo and Chinese individuals in Beijing component of HapMap as the reference [31]. 

### 2.4. Statistical Analysis

A GWAS was performed with 1,291,657 SNPs that satisfied <5% Hardy-Weinberg exact test and <5% minor allele frequency using the PLINK program. To investigate the association between the prevalence of hypertension and each SNP, the logistic multivariable regression model was applied after adjusting for age, sex, BMI, and the recruitment area of participants in the additive genetic model. A total of 178 SNPs were associated with hypertension (*p*-value < 5 × 10^−5^), and 22 tagging SNPs for 21 gene symbols (24 genes) were selected to analyze the interaction by using the linkage disequilibrium (LD) of SNPs [35].

Subjects were categorized into tertiles according to the amount of 24 h estimated urinary excretion. The general linear model (GLM) and the Cochran-Mantel-Haenzel analysis were applied to determine potential confounders. Variables with significantly different means or those that showed significant linear trends among the tertile groups were adjusted in the model as covariates. The associations of the 24 h estimated urinary factor with the risk of hypertension were examined. Multivariable logistic regression analysis was applied to calculate the odds ratios (ORs) and 95% confidence intervals (CIs) for the risk of hypertension. The interaction was evaluated by comparing models with and without interaction terms through the likelihood ratio test. All statistical analyses were conducted using SAS version 9.4 (SAS Institute, Inc., Cary, NC, USA), and PLINK [36]. 

## 3. Results

A total of 178 SNPs were associated with the risk of hypertension. Among them, 22 SNPs were selected as tagging SNPs based on high linkage disequilibrium (LD). Table 1 shows the descriptions of the 22 tagging SNPs including chromosome, position, locus, gene symbol, location, minor allele, minor allele frequency, risk allele frequency, OR, test statistic, and *p*-value.

Table 2 presents the general characteristics of the participants according to 24 h estimated urinary factors determined with the Tanaka formula. The groups with the highest 24HUNa (24 h urinary Na) and 24HUK (24 h urinary K) were older; had higher BMI and daily energy intake; had more chronic diseases (prevalence of kidney disease, obesity, or diabetes); included more non-smokers and former smokers; and had lower household income compared with the lowest group. Furthermore, the proportion of women was higher in the group with the highest 24HUK. On the other hand, the group with the highest 24HUNa-K ratio (24 h urinary Na-K ratio) was younger, and had lower BMI and daily energy intake, fewer chronic diseases, and included more current smokers and individuals with higher household incomes compared with the lowest group.

The associations of 24 h estimated urinary factors obtained with the Tanaka and Kawasaki formulas with the blood pressure are shown in Table 3. The means of SBP and DBP for the group with the highest 24HUNa were higher than those for the lowest group in all models. The means of SBP and DBP for the group with the highest 24HUK were higher than those for the lowest group in only age-sex adjusted models. The means of SBP and DBP for the group with the highest 24HUNa-K ratio were higher than those for the lowest group in multivariable models.

The associations between 24 h estimated urinary factors determined with the Tanaka and Kawasaki formulas and the risk of hypertension are shown in Table 4. Significant associations between the risk of hypertension and 24HUNa were apparent in the models using the Tanaka formula (third versus first tertile, OR = 1.21; 95% CIs = 1.00–1.48; *p*-trend = 0.037) and the Kawasaki formula (third versus first tertile, OR = 1.27; 95% CIs = 1.04–1.55; *p*-trend = 0.014). There were no significant associations between the risk of hypertension and 24HUK. However, there were significant associations between the risk of hypertension and 24HUNa-K ratio in the age-sex adjusted model and the multivariable model with the estimated Kawasaki formula (third versus first tertile, OR = 1.27; 95% CIs = 1.04–1.56; *p*-trend = 0.022).

Table 5 shows statistically significant interactions between gene polymorphisms and 24 h estimated urinary factors using the Tanaka formula. Out of 22 tagging SNPs, two SNPs (c-src tyrosine kinase (CSK) rs1378942, CSK-micro RNA 4513 (MIR4513) rs3784789) had significant interaction effects with 24 h estimated urinary factors on the risk of hypertension. The strongest protective effects of variants of CSK rs1378942 (*p*-interaction = 0.013; OR = 0.08; 95% CIs = 0.01–0.67) and CSK-MIR4513 rs3784789 (*p*-interaction = 0.027; OR = 0.08; 95% CIs = 0.01–0.72) on the risk of hypertension was found in the second tertile for the 24HUNa-K ratio.

Table 6 shows the significant interaction between gene polymorphisms and 24 h estimated urinary factors obtained with the Kawasaki formula. Among 22 SNPs, five SNPs (CSK rs1378942, CSK-MIR4513 rs3784789, uncharacterized LOC101929750 (LOC101929750) rs7554672, muskelin 1 (MKLN1) rs1643270, teneurin transmembrane protein 4 (TENM4) rs10466739) showed significant interactions between 24 h estimated urinary factors and the risk of hypertension. LOC101929750 showed the strongest risk effect for hypertension in individuals with a wild type allele who were categorized into the third tertile of 24HUNa (*p*-interaction = 0.028; OR = 2.16; 95% CIs = 1.33–3.50). The strongest protective effect was found in individuals with minor allele homozygotes of LOC101929750 who were categorized into the second tertile of 24HUK (*p*-interaction = 0.034; OR = 0.36; 95% CIs = 0.22–0.58). In the group with minor allele homozygotes of MKLN1 rs1643270, who were categorized into the second tertile of 24HUK, the strongest risk effect was observed (P-interaction = 0.034; OR = 1.55; 95% CIs = 1.01–2.38). As in Table 5 (Tanaka formula), the strongest protective effects of variants of CSK rs1378942 (*p*-interaction = 0.012; OR = 0.09; 95% CIs = 0.01–0.83) and CSK-MIR4513 rs3784789 (*p*-interaction = 0.026; OR = 0.10; 95% CIs = 0.01–0.89) were found in the second tertile of the 24HUNa-K ratio. TENM4 rs10466739 showed the strongest risk effect in individuals with heterozygotes of this gene who were in the third tertile of the 24HUNa-K ratio (*p*-interaction = 0.034; OR = 1.43; 95% CIs = 1.07–1.93).

## 4. Discussion

This study was conducted in order to identify interaction effects between gene variants and urinary sodium, potassium, and the sodium-potassium ratio on the risk of hypertension, after association analysis of hypertension with urinary factors and genetic factors. The primary finding was related to the protective effects of minor allele homozygotes of CSK rs1378942, CSK-MIR4513 rs3784789 in the middle group (second tertile) of the 24HUNa-K ratio.

There were significant positive associations of blood pressure and hypertension with 24HUNa and 24HUNa-K ratio. These results provide support that low salt diets decrease blood pressure and the risk of hypertension. With regard to 24HUK, there were no significant associations with blood pressure and hypertension. A recent meta-analysis including 14 cohort studies and 27 randomized controlled trials showed that the reduction of salt intake lowers blood pressure [18]. Other epidemiology studies showed similar results [14,15,16,17,19,20]. A possible mechanism for how sodium and potassium affect blood pressure is as follows. Salt ingestion causes increased sodium and water retention, resulting in extracellular volume expansion, which causes a release of substances that increase heart and blood vessel contraction and affect the renin-angiotensin-aldosterone system [37,38]. Potassium increases urinary sodium excretion, which decreases the amount of sodium in the body and leads to vascular smooth muscle relaxation, thereby decreasing peripheral resistance [39].

A total of 178 SNPs were significantly associated with hypertension in GWAS, and 22 SNPs for 21 gene symbols including ATP2B1 and CSK, which were frequently investigated with blood pressure and hypertension [12,40,41,42,43,44,45,46,47,48,49,50,51,52,53,54,55,56,57,58,59,60,61,62,63,64], were selected as tagging SNPs. The mutant allele of ATP2B1 rs11105368 was negatively associated with hypertension, but no significant interaction effects of urinary sodium were found in this study. The results of association between ATP2B1 and hypertension were similar to those from previous studies [10,62,63]. A recent meta-analysis study showed that the group of risk alleles, which were the same as the wild type ATP2B1 rs17249754, was significantly and positively associated with the increase of blood pressure and hypertension (β = 0.15 *p*-value = 1.75 × 10^−11^ with hypertension) [51]. The mechanism of direct influence between ATP2B1 gene variants and blood pressure has not been clearly identified, but hypotheses are continuously proposed [65,66,67]. ATP2B1 is expressed in vascular endothelium cells, and is related to the regulation of cellular calcium levels, which is known to control the contraction and expansion of vascular smooth cells [67]. ATP2B1 is related to the extrusion of Ca^2+^ [66] and the level of ATP2B1 mRNA was higher in hypertensive rats than in non-hypertensive rats [65]. 

The prominent finding of this study is the protective effect of minor allele homozygotes of CSK rs1378942 and CSK-MIR4513 rs3784789 on the risk of hypertension in the middle group for the 24HUNa-K ratio. A meta-analysis with two Korean cohorts (The KoGES_Ansan and Ansung Study and The KoGES_health examinees (HEXA) study) found that CSK increases the risk of hypertension in the group with major allele homozygotes of CSK, which is the same as the group with the risk allele [10]. CSK is known as a protein coding gene, and its molecular function is related to protein binding and protein kinase activity [68,69,70,71]. Neither the mechanism for the CSK gene variant or for blood pressure has been clearly identified, but there are meaningful studies that assume an association between CSK and blood pressure. c-Src is known to be expressed in vascular smooth muscle cells [72], to be activated by angiotensin II, and to regulate signaling action, which is associated with the migration, growth, and contraction of human vascular smooth muscle cells [73,74,75]. Although there is no direct mechanism between CSK and blood pressure, blood pressure may be associated with CSK functions related to angiotensin II and vascular smooth muscle cells. Moreover, angiotensin II is known for its role in renal sodium absorption and potassium excretion [76], which is able to affect blood pressure control. Our results proposed that there are interaction effects of CSK and urinary sodium-potassium ratio on the risk of hypertension. Overall, this study suggested that blood pressure is modified with CSK and urinary sodium-potassium ratio.

CSK-MIR4513 rs3784789 had negative associations with hypertension, and its variant had the strongest protective effects against hypertension in the middle group for the 24HUNa-K ratio. MIR4513 is a kind of micro RNA, which is a non-coding RNA, and it is related to the regulation of the expression of human genes. A recent study found effects of MIR4513 on the long-term averaging of quantitative blood pressure [77], but there is no information regarding its molecular function or a direct pathway for its relationship with blood pressure and sodium.

MKLN1 rs1643270 and TENM4 rs10466739 were positively associated with hypertension. The group with heterozygotes of MKLN1 rs1643270 had the strongest risk effects on hypertension in the middle group for 24HUK obtained with the Kawasaki formula. MKLN is one of the predicted target genes of micro RNA 27ab-3p, which is associated with smooth muscle cells [78], thus, it is possible that MKLN is associated with blood pressure control. However, this assumption has not been validated by experimental studies.

The heterozygotes of TENM rs10466739 showed the strongest risk effects on hypertension in the middle group for 24HUNa-K ratio obtained with the Kawasaki formula. TENM4 are protein coding RNA, but there were no studies or other forms of information for them related to blood pressure and sodium.

LOC101929750 rs7554672 was negatively associated with hypertension, and its variant had the strongest protective effects against hypertension in the middle group for 24HUK obtained with the Kawasaki formula. However, LOC101929750 is an uncharacterized RNA gene affiliated with a non-coding RNA, and no details or studies were found on its protein domain structure, molecular function, or pathway with blood pressure. 

Since this study used a cross-sectional design, there was a limitation in the ability to investigate the causal association between urinary factors and hypertension. Thus, further studies with independent samples are needed to confirm the interaction effects of gene polymorphisms with urinary sodium, potassium, and sodium-potassium ratio on the risk of hypertension. Among gene variants that showed interaction effects, most genes, including MIR4513, LOC101929750, MKLN1, and TENM4, have not been investigated to determine their molecular functions or pathways in blood pressure control.

There are several advantages to this study. First, to our knowledge, this study is the first large-scale investigation to identify interaction effects of 24 h estimated urinary factors (sodium, potassium, and sodium-potassium ratio) and gene polymorphism associated with the risk of hypertension through GWAS on the risk of hypertension among a Korean population. Second, possible confounding bias was minimized by adjusting various potential confounding factors (age, sex, BMI, energy intake, smoking status, regular exercise status, chronic disease prevalence, recruitment area, and household income) in the multivariable models. Third, though the use of 24 h estimation for urinary sodium using spot urine samples as a predictor of 24 h urinary excretion is still controversial, 24 h estimated urinary formulae were used instead of the spot urine test reflecting sodium intake more precisely; this method is recognized as suitable in population surveys [79].

## 5. Conclusions

In conclusion, the present study results indicate the protective effects of minor allele homozygotes of CSK rs1378942 and CSK-MIR4513 rs3784789 on hypertension in the middle group for the 24HUNa-K ratio. These findings may provide evidence for personalized nutrition to prevent hypertension. Further studies are needed to investigate the mechanisms of MIR4513, MKLN1, TENM4, and LOC101929750 on blood pressure, and to replicate these interaction effects among other populations in Korea.

## Figures and Tables

**Table 1:** Description of tagging SNPs including interaction analysis in sample 1.

SNP	CHR	Position	Locus	Gene Symbol	Location	MA	MAF	RAF	OR ^1^	STAT	*p*
rs10924160	1	243519469	1q44	KIF26B	intron	C	0.345	0.345	1.180	4.146	3.39 × 10^−5^
rs7554672	1	219339781	1q41	LOC101929750	intron	A	0.411	0.589	0.826	−4.799	1.60 × 10^−6^
rs7419838	2	38894349	2p22.1	DHX57	intron	A	0.117	0.883	0.766	−4.160	3.19 × 10^−5^
rs1997377	2	38805170	2P22.1	GALM	intron	T	0.111	0.889	0.750	−4.316	1.59 × 10^−5^
rs1562855	2	38861972	2p22.1	GEMIN6	intron	C	0.142	0.858	0.781	−4.314	1.60 × 10^−5^
rs11917719	3	24186676	3p24.2	THRB	intron	T	0.157	0.843	0.796	−4.082	4.47 × 10^−5^
rs513130	5	72456095	5q13.2	TMEM171	intron	T	0.405	0.595	0.838	−4.334	1.46 × 10^−5^
rs6457792	6	34872421	6p21.31	UHRF1BP1	intron	G	0.097	0.097	1.305	4.293	1.76 × 10^−5^
rs10260451	7	142918347	7q35	EPHA1-AS1	intron	A	0.177	0.823	0.799	−4.070	4.70 × 10^−5^
rs1643270	7	130826034	7q32.3	MKLN1	intron	C	0.479	0.479	1.197	4.569	4.90 × 10^−6^
rs3800688	7	130843015	7q32.3	PODXL	intron	G	0.419	0.419	1.197	4.600	4.23 × 10^−6^
rs16927774	8	62771785	8q12.3	ASPH	Intron	C	0.213	0.787	0.820	−4.091	4.30 × 10^−5^
rs911782	10	123996033	10q26.13	TACC2	intron	T	0.141	0.141	1.242	4.067	4.75 × 10^−5^
rs10466739	11	78290369	11q14.1	TENM4	intron	C	0.198	0.198	1.221	4.286	1.82 × 10^−5^
rs11105368	12	88598572	12q21.33	ATP2B1	Intron	C	0.376	0.624	0.810	−5.247	1.55 × 10^−7^
rs1378942	15	72864420	15q24.1	CSK	Intron	A	0.172	0.828	0.793	−4.380	1.19 × 10^−5^
rs3784789	15	72869605	15q24.1	CSK, MIR4513	Intron, upstream	G	0.169	0.831	0.799	−4.167	3.08 × 10^−5^
rs11866964	16	48217182	16q12.1	ZNF423	intron	A	0.231	0.769	0.826	−4.102	4.10 × 10^−5^
rs1858821	22	30006454	22q12.2	LIMK2	downstream	T	0.145	0.855	0.788	−4.221	2.44 × 10^−5^
rs4141404	22	30005185	22q12.2	LIMK2	3′ UTR	A	0.145	0.855	0.788	−4.221	2.44 × 10^−5^
rs2040533	22	30009110	22q12.2	PIK3IP1	missense, 3′ UTR	G	0.146	0.854	0.788	−4.211	2.54 × 10^−5^
rs2413035	22	29930460	22q12.2	RNF185	intron	T	0.149	0.851	0.791	−4.075	4.61 × 10^−5^

SNP, single nucleotide polymorphism; CHR, chromosome; MA, minor allele; MAF, minor allele frequency; RAF, risk allele frequency; OR, odds ratio; STAT, test statistic; UTR, untranslated region. ^1^ Odds ratios and *p*-values were calculated in multivariable logistic regression models, which were adjusted for age, sex, body mass index (BMI), and recruitment area.

**Table 2:** General characteristics according to 24 h estimated urinary factors obtained with the Tanaka formula in sample 2.

	Urinary Factors				
	T1 (*n* = 1471)	T2 (*n* = 1472)	T3 (*n* = 1471)	*p*-Difference	*p*-Trend
24HUNa (mEq/day)	127.8 ± 16.8 ^4^	163.4 ± 8.3	205.8 ± 29.7		
Age (years) ^1^	51.9 ± 0.2 ^a,5^	52.4 ± 0.2 ^a^	53.5 ± 0.2 ^b^	<0.0001	<0.0001
BMI (kg/m^2^) ^2^	23.8 ± 0.1 ^a^	24.5 ± 0.1 ^b^	25.3 ± 0.1 ^c^	<0.0001	<0.0001
Energy Intake (kcal/day) ^2^	1984.0 ± 21.7 ^a^	2036.3 ± 21.6 ^a, b^	2105.1 ± 21.8 ^b^	0.0004	<0.0001
Sex, women (%) ^3^	57.0 ^6^	58.2	59.0	0.580	0.299
Cigarette Smoking, current (%) ^2^	28.3	23.4	21.9	<0.0001	<0.0001
Alcohol Drinking, current (%) ^2^	41.4	44.4	42.4	0.178	0.588
Regular Exercise, yes (%) ^2^	51.6	54.6	57.9	0.003	0.001
Chronic Disease, yes (%) ^2^	34.7	45.0	54.2	<0.0001	<0.0001
Family History, yes (%) ^2^	18.3	17.9	18.7	0.887	0.753
Area, Ansan (%) ^2^	52.4	53.7	48.9	0.026	0.040
Income, ≥2,000,000 KRW (%) ^2^	31.6	31.2	25.5	<0.0001	<0.0001
24HUK (mEq/day)	33.8 ± 3.4	42.6 ± 2.5	55.6 ± 9.1		
Age (years)	50.7 ± 0.2 ^a^	52.4 ± 0.2 ^b^	54.6 ± 0.2 ^c^	<0.0001	<0.0001
BMI (kg/m^2^)	23.8 ± 0.1 ^a^	24.6 ± 0.1 ^b^	25.2 ±0.1 ^c^	<0.0001	<0.0001
Energy Intake (kcal/day)	1940.6 ± 21.5 ^a^	2031.5 ± 21.7 ^b^	2158.7 ± 22.0 ^c^	<0.0001	<0.0001
Sex, women (%)	53.1	59.7	61.6	<0.0001	<0.0001
Cigarette Smoking, current (%)	26.3	22.7	25.1	0.049	0.396
Alcohol Drinking, current (%)	43.7	41.8	42.2	0.594	0.496
Regular Exercise, yes (%)	48.4	52.3	64.1	<0.0001	<0.0001
Chronic Disease, yes (%)	35.6	45.6	53.0	<0.0001	<0.0001
Family History, yes (%)	17.9	17.8	19.8	0.404	0.226
Area, Ansan (%)	70.1	57.5	25.4	<0.0001	<0.0001
Income, ≥2,000,000 KRW (%)	35.9	30.8	20.0	<0.0001	<0.0001
24HUNa-K Ratio	3.0 ± 0.4	3.8 ± 0.2	4.8 ± 0.5		
Age (years)	53.6 ± 0.2 ^c^	52.5 ± 0.2 ^b^	51.7 ± 0.2 ^a^	<0.0001	<0.0001
BMI (kg/m^2^)	24.5 ± 0.1	24.6 ± 0.1	24.5 ± 0.1	0.781	0.815
Energy Intake (kcal/day)	2104.7 ± 21.9 ^b^	2030.4 ± 21.8 ^a^	1992.4 ± 21.5 ^a^	0.001	0.0003
Sex, women (%)	58.7	60.5	55.1	0.009	0.045
Cigarette Smoking, current (%)	27.1	23.2	23.7	0.007	0.008
Alcohol Drinking, current (%)	42.0	41.5	44.5	0.144	0.120
Regular Exercise, yes (%)	59.8	54.1	49.6	<0.0001	<0.0001
Chronic Disease, yes (%)	44.1	45.4	44.4	0.832	0.911
Family History, yes (%)	19.1	18.3	17.4	0.499	0.239
Area, Ansan (%)	31.1	54.5	70.0	<0.0001	<0.0001
Income, ≥2,000,000 KRW (%)	23.9	31.0	33.1	<0.0001	<0.0001

T, tertile; 24HUNa, 24 h urinary Na; BMI, body mass index; KRW, Korean Won; 24HUK, 24 h urinary K; 24HUNa-K Ratio, 24 h urinary Na-K ratio. ^1^ Adjusted for sex. ^2^ Adjusted for age and sex. ^3^ Adjusted for age and sex. ^4^ Mean ± standard deviation. ^5^ LS mean ± standard error for continuous variables. Values with different superscript letters within a row are significantly different means by Tukey’s multiple comparison test, *p* < 0.05. ^6^ percentiles for categorical variables.

**Table 3:** Associations between blood pressure and 24 h estimated urinary factors obtained with Tanaka and Kawasaki formulas in sample 2.

	Urinary Factors				
	T1	T2	T3	*p*-Difference	*p*-Trend
Tanaka Formula					
24HUNa (mEq/day)	127.8 ± 16.8 ^1^	163.4 ± 8.3	205.8 ± 29.7		
SBP (mmHg)					
Model 1 ^3^	116.0 ± 0.4 ^a, 2^	117.6 ± 0.4 ^b^	121.7 ± 0.4 ^c^	<0.0001	<0.0001
Model 2 ^4^	121.9 ± 1.0 ^a^	122.7 ± 1.0 ^a^	125.8 ± 1.0 ^b^	<0.0001	<0.0001
DBP (mmHg)					
Model 1	73.7 ± 0.3 ^a^	75.2 ± 0.3 ^b^	77.0 ± 0.3 ^c^	<0.0001	<0.0001
Model 2	76.6 ± 0.6 ^a^	77.6 ± 0.6 ^a^	78.6 ± 0.7 ^b^	<0.0001	<0.0001
24HUK (mEq/day)	33.8 ± 3.4	42.6 ± 2.5	55.6 ± 9.1		
SBP (mmHg)					
Model 1	117.0 ± 0.4 ^a^	117.5 ± 0.4 ^a^	120.9 ± 0.4 ^b^	<0.0001	<0.0001
Model 2	123.8 ± 1.0	122.7 ± 1.0	123.5 ± 1.0	0.182	0.718
DBP (mmHg)					
Model 1	74.4 ± 0.3 ^a^	74.8 ± 0.3 ^a^	76.7 ± 0.3 ^b^	<0.0001	<0.0001
Model 2	77.7 ± 0.7	77.1 ± 0.7	77.7 ± 0.7	0.188	0.991
24HUNa-K ratio	3.0 ± 0.4	3.8 ± 0.2	4.8 ± 0.5		
SBP (mmHg)					
Model 1	118.1 ± 0.4	118.2 ± 0.4	119.1 ± 0.4	0.188	0.093
Model 2	121.5 ± 1.0 ^a^	123.1 ± 1.0 ^b^	125.5 ± 1.0 ^c^	<0.0001	<0.0001
DBP (mmHg)					
Model 1	75.3 ± 0.3	75.1 ± 0.3	75.5 ± 0.3	0.491	0.487
Model 2	76.8 ± 0.7 ^a^	77.3 ± 0.6^a^	78.5 ± 0.6 ^b^	0.0002	<0.0001
Kawasaki Formula					
24HUNa (mEq/day)	158.0 ± 25.2	213.8 ± 13.5	286.4 ± 53.7		
SBP (mmHg)					
Model 1	116.0 ± 0.4 ^a^	118.0 ± 0.4 ^b^	121.3 ± 0.4 ^c^	<0.0001	<0.0001
Model 2	121.8 ± 1.0 ^a^	123.0 ± 1.0^a^	125.4 ± 1.0 ^b^	<0.0001	<0.0001
DBP (mmHg)					
Model 1	73.8 ± 0.3 ^a^	75.2 ± 0.3 ^b^	76.8 ± 0.3 ^c^	<0.0001	<0.0001
Model 2	76.7 ± 0.6 ^a^	77.5 ± 0.6 ^a^	78.5 ± 0.6 ^b^	<0.0001	<0.0001
Kawasaki Formula					
24HUK (mEq/day)	41.8 ± 4.6	54.0 ± 3.4	73.3 ± 14.4		
SBP (mmHg)					
Model 1	116.9 ± 0.4 ^a^	117.8 ± 0.4 ^a^	120.7 ± 0.4 ^b^	<0.0001	<0.0001
Model 2	123.6 ± 1.0	123.0 ± 1.0	123.4 ± 1.0	0.592	0.753
DBP (mmHg)					
Model 1	74.2 ± 0.3 ^a^	75.2 ± 0.3 ^b^	76.4 ± 0.3 ^c^	<0.0001	<0.0001
Model 2	77.5 ± 0.7	77.5 ± 0.7	77.5 ± 0.7	0.998	0.950
24HUNa-K ratio	2.9 ± 0.5	3.9 ± 0.3	5.2 ± 0.7		
SBP (mmHg)					
Model 1	117.7 ± 0.4	118.4 ± 0.4	119.2 ± 0.4	0.056	0.017
Model 2	121.3 ± 1.0 ^a^	123.4 ± 1.0 ^b^	125.4 ± 1.0 ^c^	<0.0001	<0.0001
DBP (mmHg)					
Model 1	75.1 ± 0.3	75.3 ± 0.3	75.6 ± 0.3	0.451	0.210
Model 2	76.6 ± 0.6 ^a^	77.6 ± 0.6 ^a,b^	78.4 ± 0.7 ^b^	0.0001	<0.0001

T, tertile; 24HUNa, 24 h urinary Na; 24HUK, 24 h urinary K; 24HUNa-K Ratio, 24 h urinary Na-K ratio; SBP, systolic blood pressure; DBP, diastolic BP. ^1^ Mean ± standard deviation. ^2^ LS mean ± standard error. Values with different superscript letters within a row are significantly different means by Tukey’s multiple comparison test, *p* < 0.05. ^3^ Model 1 was adjusted for age and sex. ^4^ Model 2 was adjusted for age, sex, body mass index, energy intake, smoking status, regular exercise status, chronic disease prevalence status, recruitment area, household income, and blood pressure medication.

**Table 4:** Odds ratios (ORs) and 95% confidence intervals (CIs) for hypertension according to 24 h estimated urinary factors obtained with Tanaka and Kawasaki formulas in sample 2.

	Urinary Factors			
	T1	T2	T3	*p*-Trend
Tanaka Formula				
24HUNa (mEq/day)	127.8 ± 16.8 ^1^	163.4 ± 8.3	205.8 ± 29.7	
Model 1 ^3^	1.00 (ref.)	1.08 (0.89–1.30) ^2^	1.59 (1.33–1.91)	<0.0001
Model 2 ^4^	1.00 (ref.)	0.93 (0.76–1.13)	1.21 (1.00–1.48)	0.037
24HUK (mEq/day)	33.8 ± 3.4	42.6 ± 2.5	55.6 ± 9.1	
Model 1	1.00 (ref.)	0.96 (0.79–1.15)	1.19 (0.99–1.43)	0.037
Model 2	1.00 (ref.)	0.79 (0.65–0.97)	0.89 (0.72–1.10)	0.400
24HUNa-K ratio	3.0 ± 0.4	3.8 ± 0.2	4.8 ± 0.5	
Model 1	1.00 (ref.)	0.94 (0.79–1.13)	1.14 (0.95–1.36)	0.161
Model 2	1.00 (ref.)	0.96 (0.79–1.18)	1.22 (0.99–1.49)	0.056
Kawasaki Formula				
24HUNa (mEq/day)	158.0 ± 25.2	213.8 ± 13.5	286.4 ± 53.7	
Model 1	1.00 (ref.)	1.22 (1.01–1.47)	1.57 (1.31–1.89)	<0.0001
Model 2	1.00 (ref.)	1.07 (0.88–1.31)	1.27 (1.04–1.55)	0.014
24HUK (mEq/day)	41.8 ± 4.6	54.0 ± 3.4	73.3 ± 14.4	
Model 1	1.00 (ref.)	0.99 (0.82–1.20)	1.14 (0.95–1.37)	0.125
Model 2	1.00 (ref.)	0.85 (0.70–1.05)	0.88 (0.71–1.09)	0.302
24HUNa-K ratio	2.9 ± 0.5	3.9 ± 0.3	5.2 ± 0.7	
Model 1	1.00 (ref.)	1.06 (0.89–1.27)	1.20 (1.00–1.44)	0.046
Model 2	1.00 (ref.)	1.11 (0.91–1.35)	1.27 (1.04–1.56)	0.022

T, tertile; 24HUNa, 24 h urinary Na; 24HUK, 24 h urinary K; 24HUNa-K Ratio, 24 h urinary Na-K ratio. ^1^ Mean ± standard deviation. ^2^ OR (95% CIs). ^3^ Model 1 was adjusted for age and sex. ^4^ Model 2 was adjusted for age, sex, BMI, energy intake, smoking status, regular exercise status, chronic disease prevalence status, recruitment area, and household income.

**Table 5:** Significant interactions between gene polymorphisms and 24 h estimated urinary factors obtained with Tanaka formula in sample 2.

	Urinary Factors			
	T1	T2	T3	*p*-Interaction
24HUNa-K Ratio				
CSK (rs1378942)				0.013
AA	1.00 (ref.)	0.08 (0.01–0.67) ^1^	0.69 (0.20–2.42)	
AC	0.61 (0.24–1.56)	0.77 (0.30–1.96)	0.67 (0.26–1.72)	
CC (wild type)	0.87 (0.35–2.16)	0.80 (0.32–2.00)	1.11 (0.44–2.76)	
24HUNa-K Ratio				
CSK-MIR4513 (rs3784789)				0.027
GG	1.00 (ref.)	0.08 (0.01–0.72)	0.77 (0.22–2.71)	
CG	0.61 (0.24–1.54)	0.74 (0.29–1.88)	0.68 (0.27–1.75)	
CC (wild type)	0.86 (0.35–2.14)	0.80 (0.32–1.99)	1.08 (0.43–2.70)	

T, tertile; 24HUNa-K Ratio, 24 h urinary Na-K ratio. ^1^ Odds ratio (95% Confidence intervals). All models were adjusted for age, sex, body mass index, energy intake, smoking status, regular exercise status, chronic disease prevalence status, recruitment area, and household income.

**Table 6:** Significant interactions between gene polymorphisms and 24 h estimated urinary factors obtained with Kawasaki formula in sample 2.

	Urinary Factors			
	T1	T2	T3	*p*-Interaction
24HUNa (mEq/day)				
LOC101929750 (rs7554672)				0.028
AA	1.00 (ref.)	1.33 (0.77–2.29) ^1^	0.79 (0.44–1.43)	
AG	1.33 (0.82–2.14)	1.25 (0.77–2.01)	1.92 (1.20–3.06)	
GG (wild type)	1.75 (1.07–2.84)	2.04 (1.26–3.31)	2.16 (1.33–3.50)	
24HUK (mEq/day)				
LOC101929750 (rs7554672)				0.034
GG (wild type)	1.00 (ref.)	0.92 (0.67–1.27)	0.72 (0.52–1.01)	
AG	0.65 (0.48–0.89)	0.58 (0.42–0.79)	0.74 (0.55–1.01)	
AA	0.67 (0.44–1.03)	0.36 (0.22–0.58)	0.40 (0.26–0.62)	
MKLN1 (rs1643270)				0.034
CC	1.00 (ref.)	1.55 (1.01–2.38)	1.22 (0.80–1.87)	
CT	1.16 (0.79–1.70)	0.83 (0.56–1.22)	0.85 (0.58–1.26)	
TT (wild type)	0.97 (0.63–1.48)	0.74 (0.48–1.14)	0.89 (0.58–1.38)	
24HUNa-K Ratio				
CSK (rs1378942)				0.012
AA	1.00 (ref.)	0.09 (0.01–0.83)	0.80 (0.23–2.75)	
AC	0.67 (0.27–1.69)	0.94 (0.38–2.35)	0.73 (0.29–1.84)	
CC (wild type)	0.93 (0.38–2.26)	0.99 (0.41–2.42)	1.25 (0.51–3.05)	
CSK-MIR4513 (rs3784789)				0.026
GG	1.00 (ref.)	0.10 (0.01–0.89)	0.89 (0.26–3.09)	
CG	0.66 (0.26–1.66)	0.91 (0.36–2.28)	0.74 (0.26–1.88)	
CC (wild type)	0.92 (0.38–2.25)	0.99 (0.40–2.42)	1.22 (0.50–2.99)	
TENM4 (rs10466739)				0.034
TT (wild type)	1.00 (ref.)	0.99 (0.77–1.26)	1.09 (0.85–1.40)	
CT	0.86 (0.63–1.16)	1.06 (0.78–1.45)	1.43 (1.07–1.93)	
CC	0.31 (0.11–0.93)	1.49 (0.78–2.85)	0.90 (0.43–1.87)	

T, tertile; 24HUNa, 24 h urinary Na; 24HUK, 24 h urinary K; 24HUNa-K Ratio, 24 h urinary Na-K ratio. ^1^ Odds ratio (95% Confidence intervals). All models were adjusted for age, sex, body mass index, energy intake, smoking status, regular exercise status, chronic disease prevalence status, recruitment area, and household income.

## Abbreviations

SNP	Single nucleotide polymorphism
GWAS	Genome wide association study
BRLMM	Bayesian Robust Linear Modeling Mahalanobis
GLM	General linear model
ORs	Odds ratios
CIs	Confidence intervals
LD	Linkage disequilibrium
24HUNa	24 h urinary Na
24HUK	24 h urinary K
24HUNa-K ratio	24 h urinary Na-K ratio

## References

[1] Mackay J., Mensah G.A. (2004). The Atlas of Heart Disease and Stroke.

[2] Alwan A. (2011). Global Status Report on Noncommunicable Diseases 2010.

[3] Ministry of Health and Welfare of Korea, Korea Centers for Disease Control and Prevention (2015). Korea Health Statistics 2014: Korea National Health and Nutrition Examination Survey (knhanes vi-2).

[4] Hong K.W., Jin H.S., Cho Y.S., Lee J.Y., Lee J.E., Cho N.H., Shin C., Lee S.H., Park H.K., Oh B. (2009). Replication of the wellcome trust genome-wide association study on essential hypertension in a korean population. Hypertens. Res..

[5] Hong K.W., Jin H.S., Lim J.E., Kim S., Go M.J., Oh B. (2010). Recapitulation of two genomewide association studies on blood pressure and essential hypertension in the korean population. J. Hum. Genet..

[6] Lee S.K., Kim S.H., Cho G.Y., Baik I., Lim H.E., Park C.G., Lee J.B., Kim Y.H., Lim S.Y., Kim H. (2013). Obesity phenotype and incident hypertension: A prospective community-based cohort study. J. Hypertens..

[7] Hong K.W., Jin H.S., Lim J.E., Cho Y.S., Go M.J., Jung J., Lee J.E., Choi J., Shin C., Hwang S.Y. (2010). Non-synonymous single-nucleotide polymorphisms associated with blood pressure and hypertension. J. Hum. Hypertens..

[8] Jin H.S., Hong K.W., Lim J.E., Hwang S.Y., Lee S.H., Shin C., Park H.K., Oh B. (2010). Genetic variations in the sodium balance-regulating genes ENaC, NEDD4L, NDFIP2 and USP2 influence blood pressure and hypertension. Kidney Blood Press. Res..

[9] Kim S.J., Lee S.K., Kim S.H., Yun C.H., Kim J.H., Thomas R.J., Shin C. (2012). Genetic association of short sleep duration with hypertension incidence—a 6-year follow-up in the korean genome and epidemiology study. Circ. J..

[10] Hong K.W., Go M.J., Jin H.S., Lim J.E., Lee J.Y., Han B.G., Hwang S.Y., Lee S.H., Park H.K., Cho Y.S. (2010). Genetic variations in ATP2B1, CSK, ARSG and CSMD1 LOCI are related to blood pressure and/or hypertension in two korean cohorts. J. Hum. Hypertens..

[11] Jin H.S., Sober S., Hong K.W., Org E., Kim B.Y., Laan M., Oh B., Jeong S.Y. (2011). Age-dependent association of the polymorphisms in the mitochondria-shaping gene, OPA1, with blood pressure and hypertension in korean population. Am. J. Hypertens..

[12] Rhee M.Y., Yang S.J., Oh S.W., Park Y., Kim C.I., Park H.K., Park S.W., Park C.Y. (2011). Novel genetic variations associated with salt sensitivity in the korean population. Hypertens. Res..

[13] Binia A., Jaeger J., Hu Y., Singh A., Zimmermann D. (2015). Daily potassium intake and sodium-to-potassium ratio in the reduction of blood pressure: A meta-analysis of randomized controlled trials. J. Hypertens..

[14] Johnson C., Raj T.S., Trudeau L., Bacon S.L., Padwal R., Webster J., Campbell N. (2015). The science of salt: A systematic review of clinical salt studies 2013 to 2014. J. Clin. Hypertens. (Greenwich).

[15] Hendriksen M.A., van Raaij J.M., Geleijnse J.M., Breda J., Boshuizen H.C. (2015). Health gain by salt reduction in europe: A modelling study. PLoS One.

[16] Sacks F.M., Svetkey L.P., Vollmer W.M., Appel L.J., Bray G.A., Harsha D., Obarzanek E., Conlin P.R., Miller E.R., Simons-Morton D.G. (2001). Effects on blood pressure of reduced dietary sodium and the dietary approaches to stop hypertension (dash) diet. Dash-sodium collaborative research group. N. Engl. J. Med..

[17] He F.J., Li J., MacGregor G.A. (2013). Effect of longer term modest salt reduction on blood pressure: Cochrane systematic review and meta-analysis of randomised trials. BMJ.

[18] Aburto N.J., Ziolkovska A., Hooper L., Elliott P., Cappuccio F.P., Meerpohl J.J. (2013). Effect of lower sodium intake on health: Systematic review and meta-analyses. BMJ.

[19] Perez V., Chang E.T. (2014). Sodium-to-potassium ratio and blood pressure, hypertension, and related factors. Adv. Nutr..

[20] Gay H.C., Rao S.G., Vaccarino V., Ali M.K. (2016). Effects of different dietary interventions on blood pressure: Systematic review and meta-analysis of randomized controlled trials. Hypertension.

[21] Whelton P.K., He J., Cutler J.A., Brancati F.L., Appel L.J., Follmann D., Klag M.J. (1997). Effects of oral potassium on blood pressure. Meta-analysis of randomized controlled clinical trials. JAMA.

[22] World Health Organization (2012). Who Guideline: Sodium Intake for Adults and Children.

[23] McLean R.M. (2014). Measuring population sodium intake: A review of methods. Nutrients.

[24] Tanaka T., Okamura T., Miura K., Kadowaki T., Ueshima H., Nakagawa H., Hashimoto T. (2002). A simple method to estimate populational 24-h urinary sodium and potassium excretion using a casual urine specimen. J. Hum. Hypertens..

[25] Kawasaki T., Itoh K., Uezono K., Sasaki H. (1993). A simple method for estimating 24 h urinary sodium and potassium excretion from second morning voiding urine specimen in adults. Clin. Exp. Pharmacol. Physiol..

[26] Mente A., O’Donnell M.J., Rangarajan S., McQueen M.J., Poirier P., Wielgosz A., Morrison H., Li W., Wang X., Di C. (2014). Association of urinary sodium and potassium excretion with blood pressure. N. Engl. J. Med..

[27] O’Donnell M., Mente A., Rangarajan S., McQueen M.J., Wang X., Liu L., Yan H., Lee S.F., Mony P., Devanath A. (2014). Urinary sodium and potassium excretion, mortality, and cardiovascular events. N. Engl. J. Med..

[28] O’Donnell M.J., Yusuf S., Mente A., Gao P., Mann J.F., Teo K., McQueen M., Sleight P., Sharma A.M., Dans A. (2011). Urinary sodium and potassium excretion and risk of cardiovascular events. JAMA.

[29] Khaw K.-T., Bingham S., Welch A., Luben R., O’Brien E., Wareham N., Day N. (2004). Blood pressure and urinary sodium in men and women: The norfolk cohort of the european prospective investigation into cancer (epic-norfolk). Am. J. Clin. Nutr..

[30] Franco V., Oparil S. (2006). Salt sensitivity, a determinant of blood pressure, cardiovascular disease and survival. J. Am. Coll. Nutr..

[31] Cho Y.S., Go M.J., Kim Y.J., Heo J.Y., Oh J.H., Ban H.J., Yoon D., Lee M.H., Kim D.J., Park M. (2009). A large-scale genome-wide association study of asian populations uncovers genetic factors influencing eight quantitative traits. Nat. Genet..

[32] Rabbee N., Speed T.P. (2006). A genotype calling algorithm for affymetrix SNP arrays. Bioinformatics.

[33] Marchini J., Howie B., Myers S., McVean G., Donnelly P. (2007). A new multipoint method for genome-wide association studies by imputation of genotypes. Nat. Genet..

[34] The Wellcome Trust Case Control Consortium (2007). Genome-wide association study of 14,000 cases of seven common diseases and 3,000 shared controls. Nature.

[35] Carlson C.S., Eberle M.A., Rieder M.J., Yi Q., Kruglyak L., Nickerson D.A. (2004). Selecting a maximally informative set of single-nucleotide polymorphisms for association analyses using linkage disequilibrium. Am. J. Hum. Genet..

[36] Plink. http://pngu.mgh.harvard.edu/~purcell/plink.

[37] Haddy F.J., Pamnani M.B. (1995). Role of dietary salt in hypertension. J. Am. Coll. Nutr..

[38] Das U.N. (2001). Nutritional factors in the pathobiology of human essential hypertension. Nutrition.

[39] Stein P.P., Black H.R. (1993). The role of diet in the genesis and treatment of hypertension. Med. Clin. North. Am..

[40] Morrison A.C., Bis J.C., Hwang S.J., Ehret G.B., Lumley T., Rice K., Muzny D., Gibbs R.A., Boerwinkle E., Psaty B.M. (2014). Sequence analysis of six blood pressure candidate regions in 4,178 individuals: The cohorts for heart and aging research in genomic epidemiology (charge) targeted sequencing study. PLoS One.

[41] Wang Y., Zhang Y., Li Y., Zhou X., Wang X., Gao P., Jin L., Zhang X., Zhu D. (2013). Common variants in the ATP2B1 gene are associated with hypertension and arterial stiffness in chinese population. Mol. Biol. Rep..

[42] Liu C., Li H., Qi Q., Lu L., Gan W., Loos R.J., Lin X. (2011). Common variants in or near fgf5, cyp17a1 and mthfr genes are associated with blood pressure and hypertension in chinese hans. J. Hypertens..

[43] Ferguson J.F., Matthews G.J., Townsend R.R., Raj D.S., Kanetsky P.A., Budoff M., Fischer M.J., Rosas S.E., Kanthety R., Rahman M. (2013). Candidate gene association study of coronary artery calcification in chronic kidney disease: Findings from the cric study (chronic renal insufficiency cohort). J. Am. Coll. Cardiol..

[44] Johnson T., Gaunt T.R., Newhouse S.J., Padmanabhan S., Tomaszewski M., Kumari M., Morris R.W., Tzoulaki I., O’Brien E.T., Poulter N.R. (2011). Blood pressure loci identified with a gene-centric array. Am. J. Hum. Genet..

[45] Van Waas M., Neggers S.J., Uitterlinden A.G., Blijdorp K., van der Geest I.M., Pieters R., van den Heuvel-Eibrink M.M. (2013). Treatment factors rather than genetic variation determine metabolic syndrome in childhood cancer survivors. Eur. J. Cancer.

[46] Wan J.P., Wang H., Li C.Z., Zhao H., You L., Shi D.H., Sun X.H., Lv H., Wang F., Wen Z.Q. (2014). The common single-nucleotide polymorphism RS2681472 is associated with early-onset preeclampsia in northern han chinese women. Reprod. Sci..

[47] Xi B., Tang W., Wang Q. (2012). Polymorphism near the ATP2B1 gene is associated with hypertension risk in east asians: A meta-analysis involving 15 909 cases and 18 529 controls. Blood Press..

[48] Heo S.G., Hwang J.Y., Uhmn S., Go M.J., Oh B., Lee J.Y., Park J.W. (2014). Male-specific genetic effect on hypertension and metabolic disorders. Hum. Genet..

[49] Fontana V., McDonough C.W., Gong Y., El Rouby N.M., Sa A.C., Taylor K.D., Chen Y.D., Gums J.G., Chapman A.B., Turner S.T. (2014). Large-scale gene-centric analysis identifies polymorphisms for resistant hypertension. J. Am. Heart Assoc..

[50] Kelly T.N., Takeuchi F., Tabara Y., Edwards T.L., Kim Y.J., Chen P., Li H., Wu Y., Yang C.F., Zhang Y. (2013). Genome-wide association study meta-analysis reveals transethnic replication of mean arterial and pulse pressure loci. Hypertension.

[51] Lu X., Wang L., Lin X., Huang J., Charles Gu C., He M., Shen H., He J., Zhu J., Li H. (2015). Genome-wide association study in chinese identifies novel loci for blood pressure and hypertension. Hum. Mol. Genet..

[52] Li J., Shi J., Huang W., Sun J., Wu Y., Duan Q., Luo J., Lange L.A., Gordon-Larsen P., Zheng S.L. (2015). Variant near FGF5 has stronger effects on blood pressure in chinese with a higher body mass index. Am. J. Hypertens..

[53] Miyaki K., Htun N.C., Song Y., Ikeda S., Muramatsu M., Shimbo T. (2012). The combined impact of 12 common variants on hypertension in japanese men, considering gwas results. J. Hum. Hypertens..

[54] Xi B., Shen Y., Reilly K.H., Wang X., Mi J. (2013). Recapitulation of four hypertension susceptibility genes (CSK, CYP17A1, MTHFR, and FGF5) in east asians. Metabolism.

[55] Hamrefors V., Sjogren M., Almgren P., Wahlstrand B., Kjeldsen S., Hedner T., Melander O. (2012). Pharmacogenetic implications for eight common blood pressure-associated single-nucleotide polymorphisms. J. Hypertens..

[56] Xi B., Zhao X., Chandak G.R., Shen Y., Cheng H., Hou D., Wang X., Mi J. (2013). Influence of obesity on association between genetic variants identified by genome-wide association studies and hypertension risk in chinese children. Am. J. Hypertens..

[57] Levy D., Ehret G.B., Rice K., Verwoert G.C., Launer L.J., Dehghan A., Glazer N.L., Morrison A.C., Johnson A.D., Aspelund T. (2009). Genome-wide association study of blood pressure and hypertension. Nat. Genet..

[58] Hotta K., Kitamoto A., Kitamoto T., Mizusawa S., Teranishi H., Matsuo T., Nakata Y., Hyogo H., Ochi H., Nakamura T. (2012). Genetic variations in the CYP17A1 and NT5C2 genes are associated with a reduction in visceral and subcutaneous fat areas in japanese women. J. Hum. Genet..

[59] Lin Y., Lai X., Chen B., Xu Y., Huang B., Chen Z., Zhu S., Yao J., Jiang Q., Huang H. (2011). Genetic variations in CYP17A1, CACNB2 and PLEKHA7 are associated with blood pressure and/or hypertension in she ethnic minority of china. Atherosclerosis.

[60] Ho J.E., Levy D., Rose L., Johnson A.D., Ridker P.M., Chasman D.I. (2011). Discovery and replication of novel blood pressure genetic loci in the women’s genome health study. J. Hypertens..

[61] Niu W., Zhang Y., Ji K., Gu M., Gao P., Zhu D. (2010). Confirmation of top polymorphisms in hypertension genome wide association study among han chinese. Clin. Chim. Acta.

[62] Takeuchi F., Isono M., Katsuya T., Yamamoto K., Yokota M., Sugiyama T., Nabika T., Fujioka A., Ohnaka K., Asano H. (2010). Blood pressure and hypertension are associated with 7 loci in the japanese population. Circulation.

[63] Fox E.R., Young J.H., Li Y., Dreisbach A.W., Keating B.J., Musani S.K., Liu K., Morrison A.C., Ganesh S., Kutlar A. (2011). Association of genetic variation with systolic and diastolic blood pressure among african americans: The candidate gene association resource study. Hum. Mol. Genet..

[64] Xi B., Shen Y., Zhao X., Chandak G.R., Cheng H., Hou D., Li Y., Ott J., Zhang Y., Wang X. (2014). Association of common variants in/near six genes (ATP2B1, CSK, MTHFR, CYP17A1, STK39 and FGF5) with blood pressure/hypertension risk in chinese children. J. Hum. Hypertens..

[65] Monteith G.R., Kable E.P., Kuo T.H., Roufogalis B.D. (1997). Elevated plasma membrane and sarcoplasmic reticulum Ca2+ pump mrna levels in cultured aortic smooth muscle cells from spontaneously hypertensive rats. Biochem. Biophys. Res. Commun..

[66] Liu L., Ishida Y., Okunade G., Shull G.E., Paul R.J. (2006). Role of plasma membrane Ca2+-atpase in contraction-relaxation processes of the bladder: Evidence from pmca gene-ablated mice. Am. J. Physiol. Cell. Physiol..

[67] Pande J., Mallhi K.K., Sawh A., Szewczyk M.M., Simpson F., Grover A.K. (2006). Aortic smooth muscle and endothelial plasma membrane Ca2+ pump isoforms are inhibited differently by the extracellular inhibitor caloxin 1b1. Am. J. Physiol. Cell. Physiol..

[68] Levinson N.M., Visperas P.R., Kuriyan J. (2009). The tyrosine kinase CSK dimerizes through its SH3 domain. PLoS One.

[69] Cloutier J.F., Veillette A. (1996). Association of inhibitory tyrosine protein kinase p50csk with protein tyrosine phosphatase pep in T cells and other hemopoietic cells. EMBO J..

[70] Autero M., Saharinen J., Pessa-Morikawa T., Soula-Rothhut M., Oetken C., Gassmann M., Bergman M., Alitalo K., Burn P., Gahmberg C.G. (1994). Tyrosine phosphorylation of CD45 phosphotyrosine phosphatase by P50CSK kinase creates a binding site for P56LCK tyrosine kinase and activates the phosphatase. Mol. Cell. Biol..

[71] Ruppelt A., Mosenden R., Gronholm M., Aandahl E.M., Tobin D., Carlson C.R., Abrahamsen H., Herberg F.W., Carpen O., Tasken K. (2007). Inhibition of t cell activation by cyclic adenosine 5′-monophosphate requires lipid raft targeting of protein kinase a type I by the a-kinase anchoring protein ezrin. J. Immunol..

[72] Oda Y., Renaux B., Bjorge J., Saifeddine M., Fujita D.J., Hollenberg M.D. (1999). Csrc is a major cytosolic tyrosine kinase in vascular tissue. Can. J. Physiol. Pharmacol..

[73] Touyz R.M., Wu X.H., He G., Park J.B., Chen X., Vacher J., Rajapurohitam V., Schiffrin E.L. (2001). Role of c-src in the regulation of vascular contraction and Ca2+ signaling by angiotensin II in human vascular smooth muscle cells. J. Hypertens..

[74] Touyz R.M., He G., Wu X.H., Park J.B., Mabrouk M.E., Schiffrin E.L. (2001). Src is an important mediator of extracellular signal-regulated kinase 1/2-dependent growth signaling by angiotensin ii in smooth muscle cells from resistance arteries of hypertensive patients. Hypertension.

[75] Mureebe L., Nelson P.R., Yamamura S., Lawitts J., Kent K.C. (1997). Activation of PP60C-SRC is necessary for human vascular smooth muscle cell migration. Surgery.

[76] Hall J.E. (1986). Control of sodium excretion by angiotensin ii: Intrarenal mechanisms and blood pressure regulation. Am. J. Physiol..

[77] Ganesh S.K., Chasman D.I., Larson M.G., Guo X., Verwoert G., Bis J.C., Gu X., Smith A.V., Yang M.L., Zhang Y. (2014). Effects of long-term averaging of quantitative blood pressure traits on the detection of genetic associations. Am. J. Hum. Genet..

[78] Albinsson S., Sward K. (2013). Targeting smooth muscle micrornas for therapeutic benefit in vascular disease. Pharmacol. Res..

[79] Hawkes C., Webster J. (2012). National approaches to monitoring population salt intake: A trade-off between accuracy and practicality?. PLoS ONE.

